# Non-Classical Intercellular Communications: Basic Mechanisms and Roles in Biology and Medicine

**DOI:** 10.3390/ijms24076455

**Published:** 2023-03-29

**Authors:** Natalia Polyakova, Maria Kalashnikova, Alexander Belyavsky

**Affiliations:** 1 Engelhardt Institute of Molecular Biology, Russian Academy of Sciences, Vavilova 32, 119991 Moscow, Russia; 2 Institute of Higher Nervous Activity and Neurophysiology, Russian Academy of Sciences, Butlerova 5A, 117485 Moscow, Russia

**Keywords:** intercellular, intercellular communications, intercellular interactions, extracellular vesicles, exosomes, microvesicles, membrane protrusions, cytonemes, tunneling nanotubes, mesenchymal stem cells, hematopoietic stem cells, oncogenesis, cell plasticity, SARS-CoV-2, COVID-19, targeted therapy

## Abstract

In multicellular organisms, interactions between cells and intercellular communications form the very basis of the organism’s survival, the functioning of its systems, the maintenance of homeostasis and adequate response to the environment. The accumulated experimental data point to the particular importance of intercellular communications in determining the fate of cells, as well as their differentiation and plasticity. For a long time, it was believed that the properties and behavior of cells were primarily governed by the interactions of secreted or membrane-bound ligands with corresponding receptors, as well as direct intercellular adhesion contacts. In this review, we describe various types of other, non-classical intercellular interactions and communications that have recently come into the limelight—in particular, the broad repertoire of extracellular vesicles and membrane protrusions. These communications are mediated by large macromolecular structural and functional ensembles, and we explore here the mechanisms underlying their formation and present current data that reveal their roles in multiple biological processes. The effects mediated by these new types of intercellular communications in normal and pathological states, as well as therapeutic applications, are also discussed. The in-depth study of novel intercellular interaction mechanisms is required for the establishment of effective approaches for the control and modification of cell properties both for basic research and the development of radically new therapeutic strategies.

## 1. Introduction

In modern biomedicine, the emergence of new cell therapy approaches potentially capable of significantly expanding the possibilities of classical pharmacology determines the increasing interest in the deep transformation of cellular properties. Particular attention is now being paid to the study of intercellular interactions and communications, which, for example, are primarily responsible for the therapeutic effects of stem cells (SCs) and the phenomenon of education of normal cells by tumors in vitro and in vivo [1,2,3].

Four types of communications by animal cells are universally accepted, namely, endocrine, paracrine and contact signaling, as well as nervous transmission [4,5,6,7,8]. Endocrine signaling is mediated by the production of hormones in the endocrine glands and their systemic distribution to distant target cells. Paracrine signaling has a narrower range and is mediated by the local action of paracrine signaling substances secreted by cells into intercellular spaces. In contact signaling, cell surface molecules bind to a receptor on a neighboring cell, while nerve signal transmission is carried out along axons to distant target cells. In this review, by classical interactions we primarily mean well-known interactions of the ligand–receptor type; direct intercellular adhesive contacts, including desmosomes and related contact types; gap junctions providing ionic conjugation of cells; as well as synapses formed between cells of excitable tissues. Over the past decade, however, new, relatively little-studied types of intercellular communication have significantly gained in importance within the scientific community. More and more facts are being discovered about the crucial role in biological processes of non-classical interactions mediated by extracellular microvesicles as well as various membrane protrusions.

Since extracellular vesicles—in particular, exosomes—have significant biotechnological potential but are also involved in pathogenesis, the study of the mechanisms underlying the maturation and release of exosomes may be important for the development of new therapeutic approaches in medicine. In preclinical trials, the effects of microvesicles and exosomes obtained from conditioned stem cell media are comparable to the regenerative effects obtained with SC transplantation [9,10,11]. At the same time, the clinical use of stem cells themselves is still associated with problems resulting primarily from their low survival rate after transplantation [12,13].

In addition, the transfer of extracellular vesicles using other non-classical methods of intercellular communication considered in this review, namely, through cytonemes and tunneling nanotubes, is of considerable interest, since the ability of exosomes and microvesicles to move along the formed “bridges” can be used in the future for targeted vesicle delivery.

## 2. Extracellular Vesicles

The origins of extracellular vesicle research dates back to the forties of the last century when experiments with blood coagulation revealed that platelets produce a clotting factor. Subsequently, this factor was identified as a constituent of small vesicles 20–50 nm in size secreted by platelets [14,15,16]. In 1981, the term “exosomes” was first proposed, when electron microscopic studies of various cell lines revealed vesicles surrounded by a lipid membrane of two size classes: about 40 nm in diameter and 500–1000 nm in diameter [17]. Several decades later, despite the fact that there was still no complete agreement on the nomenclature of vesicles, the term “extracellular vesicles” (EVs) was proposed to be used in relation to all secreted vesicles [18]. At present, the “Minimum Information for Studies of Extracellular Vesicles” (MISEV) was proposed by The International Society for Extracellular Vesicles (ISEV) as a universal guide for the classification of extracellular vesicles, as well as non-vesicular nanoparticles (exomers) [19].

The criteria for the classification of vesicles include various biochemical and functional characteristics, such as size, morphology, density, lipid composition, protein composition and subcellular origin. Two main classes of EVs are commonly recognized, namely, exosomes (30–150 nm) and microvesicles/ectosomes (100–1000 nm). However, exemplifying the great diversity and heterogeneity of EVs, several other, larger but less common types of vesicles have been described, including apoptotic bodies (0.8–5 μm) released by apoptotic cells and large vesicles (1–10 μm) released by various cells, called oncosomes in the case of tumor cells [20,21,22].

### 2.1. Exosomes as a Special Class of Extracellular Vesicles: Biogenesis, Molecular Composition and Distribution

Exosomes are secreted by a variety of cell types, including but not limited to stem, dendritic, B- and T-cells, endothelial cells, cardiomyocytes, Schwann cells, platelets, and tumor cells [23,24,25,26,27,28,29,30,31], and play an essential role in modulating immune responses, as well as cellular signaling pathways [32,33]. Besides their reported role in a myriad of biological processes, exosomes are apparently the most intensely secreted class of extracellular vesicles. Thus, according to the estimates of one study [34], the CHO cell produces on average 203 exosomes and 68 microvesicles per day.

**Figure 1:**
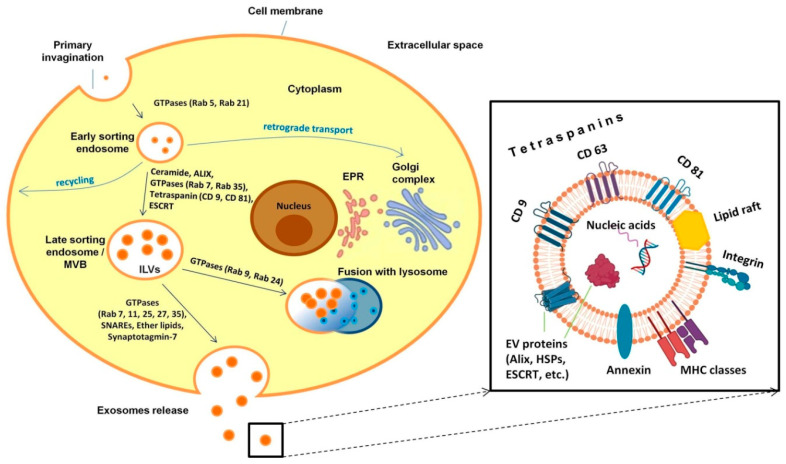
Schematic presentation of exosome formation, release and molecular composition. The early sorting endosome forms as a result of primary invagination under the control of GTPases, including Rab5 and Rab21. The maturation of an early endosome into a late sorting endosome involves such factors as ceramides, ALIX, Rab7, Rab35, tetraspanins (CD9 and CD81), as well as endosomal sorting complexes required for transport. The fusion of multivesicular bodies with lysosomes and their degradation occurs under the control of the GTPases Rab9 and Rab24. The fusion of multivesicular bodies with the cell membrane followed by the release of exosomes into the extracellular space is mediated by Rab family GTPases, SNAREs (soluble NSF attachment receptors) and other elements. Mature exosomes are spherical formations surrounded by a two-layer lipid membrane enriched with ceramides, cholesterol, sphingomyelin, phosphatidylserine and glycosphingolipids. The exosome molecular cargo can include various families of proteins, adhesion molecules, receptors, various RNAs and DNA fragments. ILVs—intraluminal vesicles, MVBs—multivesicular bodies, EPR—endoplasmic reticulum, ESCRT—endosomal sorting complexes required for transport, SNAREs—soluble N-ethylmaleimide-sensitive factor attachment protein receptors, HSPs—heat shock proteins, MHC—major histocompatibility complex.

Exosome biogenesis is distinct from that of the other extracellular vesicles that are released from the plasma membrane and is a complex process that includes invagination of the plasma membrane with the formation of intracellular multivesicular bodies (MVBs) [20]. A schematic representation of the pathway leading to exosome formation is shown in Figure 1.

Primary invagination of the plasma membrane results in the formation of an early sorting endosome (ESE). At this stage, various molecular “cargoes”, including solutes, lipids, receptors and pathogenic agents, enter the cell as part of the endocytic process. Importantly, the effective sorting of the material that has entered the cell is a key part of the normal cell function affecting a host of processes, such as signal transmission, cell metabolism, cell growth and protective reactions, to name a few.

The maturation of the early endosome and its movement from the periphery towards the cell nucleus are accompanied by the formation of elongated tubular sections, as well as areas with invaginations of the inner membrane within the endosome. During the initial stage of sorting in the early endosome, some of the molecules return back to the cell surface during recycling as part of tubular structures, some of the molecules are delivered to the trans-Golgi network via retrograde transport, and the multivesicular part of the early endosome subsequently transforms into the late sorting endosome (LSE) [35].

The formation of multivesicular bodies (MVBs) containing prospective exosomes (called at this stage intraluminal vesicles (ILVs)) occurs due to internal invagination of the endosomal membrane. A distinctive feature of this stage is the reorganization and enrichment of the endosomal membrane with transmembrane proteins, tetraspanins, which are the main markers of exosomes (CD9, CD63 and CD81) and are involved in sorting the molecular cargo of exosomes [36,37,38].

To date, several mechanisms of IV formation have been described. One of the most important ones recruits the ESCRT (the endosomal sorting complexes required for transport) towards the place of formation of IVs. A number of studies have demonstrated a clear division of functions between ESCRT complexes. Thus, the ESCRT-0 heterodimer recognizes ubiquitinated proteins outside the endosomal membrane and is responsible for cargo clustering. It forms sorting microdomains and recruits ESCRT-I and ESCRT-II complexes, which predominantly induce membrane invagination and attract the Alix protein. ESCRT-I binds the ubiquitinated cargo on endosomes, resulting in the activation of ESCRT-II, which is responsible for the budding of the intraluminal membrane. The Alix protein recruits the ESCRT-III complex, which buds off and separates intraluminal vesicles [36,39,40]. The second mechanism for the formation of intraluminal vesicles is not associated with ESCRT and protein ubiquitination and involves instead the oligomerization of the transmembrane protein syndecan and its binding to syntenin, which leads to the recruitment of CD63 and Alix and the formation of intraluminal vesicles [30]. The third mechanism of intraluminal vesicle formation, studied in less detail, is associated with a shift in the lipid composition of the endosomal membrane, resulting in lipid clustering into specific subdomains, rafts, which induce membrane invagination with subsequent formation of vesicles [41].

The subsequent fate of mature MVBs implies two possible outcomes: fusion with lysosomes or autophagosomes, ultimately leading to degradation of the contents, and fusion with the cell membrane to release the intraluminal vesicles as exosomes. The release of exosomes into the intercellular space is controlled by GTPases of the Rab family—in particular, Rab11, Rab27 and Rab35. Rab11 and Rab35 regulate the recycling of membrane components from the endosome to the plasma membrane. Rab27 is involved in the transport of late endosomes and lysosome-like compartments to the plasma membrane. Rab27, together with the secretory GTPase Rab3 and recirculating Rab11, is responsible for the regulation of sequential exocytic events [42,43,44].

Ceramides, a special type of lipid molecules of the sphingolipid class, which are formed from sphingomyelins under the action of neutral sphingomyelinases (N-SMase 2) also play a significant role in the formation and secretion of exosomes [45]. Due to the cleavage of sphingomyelins by sphingomyelinases, the local level of ceramides in the membrane increases, resulting in its budding [46].

Mature exosomes are spherical formations surrounded by a bilayer lipid membrane, the inner layer of which is enriched with ceramides. Exosome membranes are also enriched in cholesterol, sphingomyelin, phosphatidylserine and glycosphingolipids [47]. The bilayer membrane endows exosomes with high stability, contributing to the preservation of their cargo. The molecular composition of exosomes largely depends on the physiological state of the cell and its functions, as well as on environmental factors that the cell is exposed to (for example, hypoxia) [48,49]. The molecular cargo of exosomes can include various families of proteins (annexins, integrins, heat shock proteins (HSPs), proteins of the major histocompatibility complex (MHC) and ESCRT), adhesion molecules, receptors, microRNAs, messenger RNAs and DNA fragments [50].

The study of Obata et al. [51] revealed that adiponectin, a protein hormone from adipocytes, enhances exosome biogenesis in a T-cadherin-dependent manner. Accordingly, expression of T-cadherin was accompanied by a significant increase in exosome production, while knockdown of T-cadherin had an opposite effect. Another study demonstrated the regulation of exosome secretion by the MAL protein [52], which, in addition to other functions, is necessary for the transport of proteins to the plasma membrane and the organization of the immunological synapse in human T cells [53]. MAL has been shown to be required for the efficient fusion of endosomes with the plasma membrane and release of the contents of intraluminal vesicles into the extracellular space, as well as for the correct sorting of exosomal cargoes in MVBs [52]. Gurunathan et al. [54] studied the effect of platinum nanoparticles on biogenesis and the release of exosomes in A549 lung epithelial adenocarcinoma cancer cells. Cultivation of A549 cells with these nanoparticles resulted in increased secretion of exosomes, which was caused by the induction of oxidative stress and the ceramide pathway—factors that enhance exosome biogenesis [54].

Finally, an unconventional pathway for exosome formation is described in a new study by Arya et al. [55]. The biogenesis of exosomes containing a secondary neutrophil chemoattractant, leukotriene B4 (LTB4), begins in the nuclear membrane of neutrophils. At the same time, the generation of ceramide-rich lipid microdomains mediated by neutral sphingomyelinase N-SMase1 contributes to the clustering of enzymes synthesizing LTB4 (5-lipoxygenase (5-LO), 5-5-LO-activating protein (FLAP) and LTA4 hydrolase) on the nuclear shell. The authors suggested that the initiation of nuclear envelope curvature followed by budding is mediated by the N-SMase1-dependent generation of ceramides, leading to the recruitment of FLAP and ALIX [55].

### 2.2. Extracellular Vesicles as Potential Therapeutic Agents

It is firmly established that, following systemic administration, mesenchymal stem/stromal cells (MSCs) survive in the body for only a very short time. Therefore, it is likely that immunomodulation and organ regeneration attributed to MSCs are in many instances due to the secretion of extracellular vesicles by these cells. Recent studies have demonstrated significant therapeutic effects achieved with extracellular vesicles produced by MSCs. For example, MSC-derived vesicles can be used to treat chronic skin ulcers (CSUs) arising due to metabolic diseases or aging. Preclinical studies have shown a significant improvement in CSU healing and reduction in scarring with extracellular MSC-derived EVs [56]. These results were mediated by the immunosuppressive and immunomodulating properties of EVs, the activation of angiogenesis, and the proliferation, migration and differentiation of the main cell types involved in regeneration—endothelial cells, fibroblasts and keratinocytes. Another work demonstrated that EVs secreted by bone marrow MSCs markedly reduce acute lung injury in mice by delivering keratinocyte growth factor (FGF7) mRNA [57]. In addition, extracellular vesicles were shown to be involved in the processes of neuroregeneration and neurological recovery, as well as in the modulation of peripheral post-stroke immune responses [58]. Liu et al. [59] demonstrated that the administration of exosomes derived from bone marrow mesenchymal stem cells (BMSC-exos) into the lateral ventricle, but not the caudal vein, improved the behavioral performance of mice in a model of Alzheimer’s disease. A new potential alternative to the systemic treatment of keloids was proposed by Li et al. [60], who studied the effect of exosomes from human adipose tissue MSCs (adMSC-Exos) on extracellular matrix (ECM) remodeling in keloids using in vitro and ex vivo models. Promising results regarding the induction of chondrogenesis and the treatment of osteoarthritis with exosomes isolated from Wharton’s jelly MSCs were obtained by Chen et al. [61]. In particular, the authors showed that, in these exosomes, let-7e-5p miRNAs regulating the production of the transcription factor STAT3 and insulin-like growth factor receptor-1 (IGF1R) serve as active components for cartilage regeneration. Another group of researchers determined that the circular RNA circPARD3B associated with extracellular vesicles is a new marker of osteoarthritis and also demonstrated that EVs from synovial MSCs overexpressing circPARD3B can postpone disease progression, paving the way for the development of new methods of treating osteoarthritis [62]. Another approach has been proposed as an alternative to autologous nerve transplantation in the treatment of peripheral nerve lesions [63]. In this study, PKH26-labeled iPSC-derived exosomes in combination with acellular nerve grafts (ANGs) were used to bridge 15 mm peripheral nerve gaps in a model of motor dysfunction in rats with positive dynamics and restoration of motor function. According to one of the latest meta-analyses of the use of extracellular vesicles from MSCs in the treatment of cerebral ischemia, these vesicles can significantly reduce neurological disorders, as well as help reduce the volume of cerebral infarction and reduce neuronal apoptosis by inhibiting microglia-mediated neuroinflammation [64]. Finally, a new, promising approach based on the specific sorting of effective exosomes has been proposed recently [65]. According to this work, sorted exosomes containing encapsulated therapeutic Ldlr-MS2 mRNA had a significantly higher efficiency in reducing atherosclerosis compared to unsorted ones.

Among the works aimed at the therapeutic use of exosomes, considerable attention has been paid to anti-aging effects. A very important recent study showed that extracellular vesicles derived from MSCs from young mice can slow down aging and increase the lifespan of mice [66]. One of the first external signs of aging is the deterioration of the epidermis, and thus the attempts to use cells and cellular products for skin rejuvenation attract much attention. The study by Guo et al. revealed substantial anti-aging effects of exosomes from ADSCs (ADSC-exos) on human dermal fibroblasts (HDFs)—in particular, increased expression of type I collagen—as well as reductions in reactive oxygen species (ROS) and aging-associated β-galactosidase (SA-β-Gal) levels. In addition, there was a significant decrease in the levels of p16, p21 and p53 proteins associated with aging and cell senescence, as compared to the control group [67].

Ma et al. [68] determined that ADSC-exos had stimulatory effects on the proliferation and migration of human keratinocytes (HaCaTs) and could also suppress apoptosis, showing increased expression of β-catenin and activation of Wnt/β-catenin signaling. In an earlier study, Kim et al. [69] demonstrated that the effects of exosomes isolated from induced MSCs (iPSCs-derived MSCs) and intact MSCs from Wharton’s jelly on the proliferation of keratinocytes and dermal fibroblasts were comparable. Recent experiments in a model of acute photodamage and skin aging revealed that subcutaneous injection of exosomes derived from human umbilical cord MSCs (hucMSC-exos) had anti-inflammatory and antioxidant effects, protecting skin cells from DNA damage and apoptosis. The cytoprotective effect of hucMSC-exos was mediated by the delivery of 14-3-3ζ protein and modulation of the SIRT1-dependent antioxidant pathway [70]. In a very recent study, Natale et al. [71] demonstrated that insulin resistance induces and accelerates the aging process of the neurogenic niche by epigenetically stimulating the expression of the inhibitor of cyclin-dependent kinase 1 (p21). Stimulation of neural stem and progenitor cells (NSPCs) by NSPC-derived exosomes rescued IRS-1/FoxO activation and counteracted stem cell aging. Moreover, intranasal administration of exo-NSPCs reduced the high-fat diet-dependent impairment of adult hippocampal neurogenesis in mice. The results of this study may pave the way for the development of novel therapies that reduce neurocognitive impairments during aging.

Summarizing, the above results show that EV-based therapy, including MSC-derived vesicles, is a promising tool for the treatment of a number of diseases. Various preclinical studies indicate that EVs produced by stem cells are able to stimulate the repair and regeneration of tissues, organs and organ systems, including the nervous, cardiovascular, respiratory and musculoskeletal systems. At the same time, exosome administration seems to largely lack the negative side effects potentially associated with direct stem cell transplantation, such as immune reaction and low engraftment.

### 2.3. Extracellular Vesicles and Coronavirus Infection

Results obtained in the last few years indicate that EVs play a significant role in the pathogenesis of coronavirus infection caused by SARS-CoV-2. One of the main receptors for SARS-CoV-2 in the human body is angiotensin-converting enzyme 2 (ACE2) [72]. A 2020 study showed the presence of ACE2 in exosomes and the EV-mediated transfer of ACE2 between different cells [73]. According to another study, circulating exosomes in patients infected with respiratory viruses contain viral antigens [74]. Thus, intercellular communication via EVs can accelerate the development of infection by transferring individual constituents of SARS-CoV-2 and viral particles to susceptible cells.

Dysregulation of the immune response, leading to cytokine storm, is one of the reasons for the high mortality of patients with acute respiratory distress syndrome (ARDS) in severe cases of COVID-19 [75]. Therefore, elucidating the role of EVs in cytokine storm during coronavirus disease is an important area of contemporary research. Evaluation of the number of EVs in patients with ARDS and cytokine storm detected higher concentrations of EVs in patients with ARDS in pulmonary edema fluid relative to the control group, namely, patients who had hydrostatic pulmonary edema [76]. In mouse experiments, exosome secretion, as well as exosome-associated protein production, was higher in the lungs of asthmatic animals compared to control mice. At the same time, the use of the neutral sphingomyelinase inhibitor GW4869 led to the suppression of exosome production, a decrease in the population of proliferating monocytes and alleviation of asthma manifestations [77].

Despite the pathological role of extracellular vesicles in the spread of the virus throughout the body and the initiation of cytokine storm, extracellular vesicles—in particular, MSC exosomes—are now considered promising therapeutic agents in the treatment of COVID-19 [78]. Khanh et al. [79] showed that extracellular MSC vesicles reduced the levels of inflammatory cytokines regulated by the SARS-CoV-2 peptide in Calu-3 cells. A clinical, non-randomized study using exosomes of allogeneic bone marrow MSCs (ExoFlo™) demonstrated significant hypoxia reversal, cytokine storm suppression and immune recovery in patients who were hospitalized with severe COVID-19 [80]. Another study established that MSC-derived EVs contributed to the suppression of pro-inflammatory reactions in SARS-CoV-2-infected cells and cell recovery after cytopathic action by inhibiting virus replication [81]. Antiviral responses were mediated by several miRNAs, five of which were complementary to conserved 3′-untranslated regions (UTRs) of the viral genome, which led to suppression of SARS-CoV-2 replication.

More recently, a novel therapy aimed at neutralizing SARS-CoV-2 has been described that employs EVs engineered to contain the tetraspanin protein CD63 embedded within an anti-CoV-2 nanobody. These vesicles bind the SARS-CoV-2 spike protein in the region of the receptor-binding domain and thus could efficiently neutralize SARS-CoV-2 [82].

## 3. EV-Mediated Intercellular Interactions in Stem Cell Biology and Plasticity

Since, in the previous part of this review, the therapeutic effects of stem cells mediated by non-classical intercellular interactions have been considered, it would be appropriate to discuss now the role of these types of intercellular communication in the biology of stem cells themselves.

### 3.1. Extracellular Vesicles in the Bone Marrow Niche as Regulators of HSC Quiescence, Expansion and Differentiation

In maintaining homeostasis and regulating the functions of many, if not all, types of stem cells (SCs), the so-called niche plays a decisive role, the specific components of which create a favorable and carefully controlled environment ensuring the survival and proper functioning of SCs. The bone marrow, along with hematopoietic stem cells (HSCs) and progenitors, as well as endothelial cells, also contains MSCs and cells derived from MSCs, such as osteoblasts and adipocytes, which, in various combinations and with the participation of the extracellular matrix, constitute a specialized niche that regulates the state and functioning of HSCs [83]. Back in the 1960s, MSCs were discovered as non-hematopoietic cells of the bone marrow microenvironment supporting the process of hematopoiesis [84]. The interaction between MSCs and HSCs controls their differentiation and protects them from apoptosis, thus promoting the self-renewal and maintenance of HSCs [85,86].

Although the roles of paracrine signaling and adhesive interactions in HSC-niche intercellular interactions are firmly established (see, for example [83,87,88,89]), the roles of various types of non-classical communications remain to be elucidated. Nevertheless, it has been reliably established that a significant role in the modulation of the bone marrow niche is played by extracellular vesicles that can change the biology of HSCs and progenitor cells [90,91]. Ex vivo studies show that MSC microvesicles contain effector molecules, including Wnt and Hedgehog morphogens, which regulate SC self-renewal, proliferation and differentiation [92,93]. Moreover, microRNAs of MSC vesicles are actively involved in the regulatory processes in HSCs. In particular, these vesicles contain miRNAs that suppress the production of many Wnt inhibitors in HSCs and precursors, which, in ex vivo cultures, leads to an increase in the number of colony-forming units [94]. EVs from MSCs also increase CXCR4 expression in cord blood HSCs, stimulating their homing to the bone marrow [95]. Finally, the role of MSC vesicles in the regulation of hematopoiesis through innate immune mechanisms has been identified. In particular, MSC microvesicles are able to stimulate the expansion of HSCs and their myeloid differentiation through interaction with TLR4 (Toll-like receptor 4) [96].

A study by Salvucci et al. [97] found that granulocyte colony stimulating factor (G-CSF) promotes the accumulation of miR126-containing vesicles in the bone marrow extracellular compartment. Vesicle-delivered miR126 reduces surface expression of VCAM1 in HSCs and progenitor, stromal and endothelial cells, thus regulating the mobilization and movement of HSCs and progenitor cells between the bone marrow and peripheral sites. Another study established that exosomes produced by IL-4-polarized M2 macrophages have an immunomodulatory function and are able to limit the expansion of hematopoietic progenitors and reduce inflammation [98].

In recent years, considerable attention has been paid to the aging of the hematopoietic system, studied primarily in mouse models [99,100]. Recent data show both the role of hematopoietic niches in aging and the participation of extracellular vesicles in this process. In particular, H_2_O_2_ levels in the bone marrow microenvironment have been shown to increase with age, while treatment of bone marrow stem cells with H_2_O_2_ increased the amount of miR-183-5p in extracellular vesicles. This in turn caused a decrease in cell proliferation and aging, as well as reduction in heme oxygenase 1 levels (Hmox1) [101]. In addition, endocytosis of miR-183-5p+ vesicles by young bone marrow stromal cells leads to inhibition of their proliferation and suppression of osteogenic differentiation.

It should be noted that the effects of extracellular vesicles on the aging of the human hematopoietic system, in contrast to the murine system, have been little studied. Grenier-Pleau et al. [102] characterized blood EVs in people aged 20–85 years, demonstrating that while EVs’ external parameters, such as size, were constant with aging, the protein profiles of EVs did change. As a result, EVs from donors older than 40 years stimulated HSCs, in contrast to the EVs from younger persons. A new study [103] examined the modulation of cell cycle activity and clonogenicity of HSCs by extracellular vesicles. EVs from young MSCs were able to support the expansion of HSCs in vitro, while vesicles from old MSCs did not have a positive effect on cell survival and proliferation. This was accompanied by changes in gene expression, since in HSCs treated with vesicles of old MSCs, a decrease in the expression of the tumor suppressors PTEN and CDKN2A was observed. The above data are in good agreement with the notion that aging is associated with the risks of developing cancer and is caused by serious changes in the functions and phenotypes of cells, induced, among other things, by horizontal or epigenetic regulation of cell life processes.

### 3.2. Extracellular Vesicles in Oncogenesis

Leukemic exosomes have various effects on the viability and differentiation of HSCs. Thus, exosomes of acute myeloid leukemia are able to change the function of HSCs or bone marrow stromal cells, transferring miR-155 to HSCs and participating in slowing down their differentiation due to a decrease in c-MYB expression [104]. In chronic lymphocytic leukemia, microvesicles activate the AKT pathway and elevate the production of hypoxia-induced factor 1-alpha (HIF-1α) and vascular endothelial growth factor (VEGF), changing the bone marrow niche in a malignant direction [105].

A large number of data have convincingly demonstrated that EVs of tumor cells have a significant effect on the development of tumors—in particular, they recruit and remotely modify normal body cells in the pre-metastatic niche during metastasis and induce angiogenesis and other processes associated with tumor growth [106,107,108]. Thus, the primary tumor has the ability to selectively modify the microenvironment of distant organs before metastasis, and both vesicles of tumor origin and bone marrow cells are involved in this process [109,110,111].

Currently, active research is being conducted in the field of hematopoiesis suppression in multiple myeloma [112,113,114,115]. The critical role of EVs in the pathogenesis of multiple myeloma was demonstrated in a recent study where extracellular vesicles derived from multiple myeloma cells (MM-EVs) transmitted oncogenic NOTCH2 receptors and increased NOTCH signaling in distant targets, affecting the pro-tumorogenic behavior of endothelial cells and osteoclast precursors [116]. Another study established that MM-EVs activate proliferation and induce an increase in the frequency of HSCs and early progenitors, with a simultaneous decrease in the frequency of later progenitors and impaired colony formation by HSPCs [117]. These data are in good agreement with the recent findings of Lopes et al. [118] that MM-EVs are able to modulate the immune microenvironment of the bone marrow, thereby changing the expression of key factors involved in the regulation of the antitumor activity of T cells (for example, IC PD-1 and CTLA-4).

The pathological effect of tumor-derived EVs is mediated by their molecular cargo, which differs significantly from that of normal ones. Thus, exosomes from the plasma of cancer patients contain such markers as PD-1/PD-L1, TRAIR/TRAIL and Fas/FasL [119]. The permanent components of tumor exosomes also include miRNAs, oncogenic DNA sequences (HRAS, BCR-ABL and KRAS), cell adhesion molecules, as well as other proteins [120]. In metastatic prostate cancer, for example, tumor extracellular vesicles migrate to the bone, where the absorption of miR-378a-3p from these vesicles by bone marrow macrophages initiates the process of osteolysis along the Dyrk1a/Nfatc1 pathway, which in turn contributes to an increase in Angptl2 secretion and tumor progression [121].

The key role of extracellular vesicles in communication between adipocytes and breast cancer cells was shown in a recent study describing a new mechanism that enhances the malignant potential of breast cancer cells [122]. The metastatic effects of extracellular vesicles were mediated by the induction of HIF-1α and could be neutralized by the HIF-1α inhibitor KC7F2 or suppression of its expression. Interestingly, extracellular vesicles from undifferentiated adipocytes did not induce HIF-1α expression and did not contribute to tumor progression.

Among other things, exosomes and microvesicles of tumor cells are capable of reprogramming mesenchymal stem cells into cells supporting tumors and even tumor-like cells [123,124,125,126]. Thus, tumor exosomes carrying TGF-β1 and VEGF induced reprogramming of MSCs into pro-invasive and pro-angiogenic myofibroblasts [127,128,129]. Gyukity-Sebestyén et al. [123] showed that MSCs in the process of communication with metastatic melanoma cells via exosomes are subject to malignant reprogramming that converts them into melanoma-like, PD-1 overexpressing cell populations. This work identified a tumorigenic protein signaling network that includes such key regulators as RAF1, MET, BCL2, PD-1 and mTOR in MSCs treated by exosomes. These results correlate with earlier findings by Kleffel et al. [130] that in aggressive melanoma populations, high expression of PD-1 and activation of the mTOR signaling pathway contributed to tumor progression, while inhibition of PD-1 signaling attenuated melanoma aggressiveness and metastasis. In another study, the effects of exosomes from K562 chronic myeloid leukemia cells on the gene expression, cytokine secretion and redox potential of bone marrow MSCs and macrophages have been convincingly demonstrated [131]. Accumulated data indicate that, when exposed to tumor vesicles, MSCs acquire a significant number of functions characteristic of cancer cells, including migration to the tumor site, production of pro-inflammatory cytokines, stimulation of angiogenesis and tumor growth, induction of the epithelial-mesenchymal transition and suppression of immune effector cells [132,133,134]. The reprogramming of MSCs by extracellular vesicles of tumor origin is thus a continuous process serving the needs of growing and metastasizing tumors.

On the other hand, many antitumor effects are also known to be exerted by vesicles of MSCs and other stem cells. Phetfong et al. [135] recently established that BM MSC EVs suppressed cell proliferation and induced apoptosis of chronic myeloid leukemia (K562) and acute promyelocytic leukemia (NB4) cells in vitro. According to one of the latest meta-analyses, MSC-EVs had a mixed response to tumor progression. However, significant suppression of tumor growth was observed frequently with vesicles from MSCs that overexpressed anti-tumor RNA [136]. Finally, the efficiency of antitumor therapy can be increased by using exosomes derived from osteogenic differentiating MSCs, which induce osteogenic differentiation of cancer stem cells and reprogram them into non-tumor cells [137].

### 3.3. The Use of Extracellular Vesicles for the Induction of Plasticity and Transdifferentiation of Cells

Adult resident SCs are normally able to differentiate into several different cell types, which is vital for tissue homeostasis [138]. In this sense, SCs already have an inherent plasticity potential, in contrast to mature differentiated cells, which are expected to maintain their specific identities. However, the plasticity of SCs and other cells, in the true sense, implies substantial expansion of their differentiation potential beyond what is available to them normally.

The substantial effect of EVs on the plasticity of SCs and other cells was first established in the laboratories of M. Ratajczak [139] and P. Quesenberry [140]. In one study [139], microvesicles of embryonic SCs were demonstrated to significantly increase survival and stimulate the expansion of hematopoietic progenitors, as well to increase the expression of the pluripotent markers Oct4, Nanog, and Rex-1 and the early hematopoietic markers Scl, HoxB4, and GATA 2. Another study [140] revealed that microvesicles of cells from radiation-damaged lungs can affect bone marrow cells, causing them to express genes characteristic of lung epithelial cells, as well as increase their ability to produce type 2 pneumocytes after transplantation. Subsequent studies by the Quesenberry laboratory showed that the described effects result in long-term and stable transcriptome changes [141]. Moreover, these effects were dependent on the stage of the cell cycle [142].

A striking example of stem cell plasticity mediated by exposure to EVs is the recent work on the differentiation of human adipose stem cells (HASCs) into white and beige adipocytes, which was induced by vesicles isolated from HASCs during adipogenic differentiation into the different adipocyte types [143]. These experiments demonstrated the presence of factors in EVs that promote the differentiation of stem cells in one direction or another in vitro and in vivo.

The potential of extracellular vesicles in cell transdifferentiation was convincingly shown in a recent study [144], where a very high efficiency of reprogramming fibroblasts into functional cardiomyocytes was achieved by using vesicles from embryonic SCs in which cardiomyogenic differentiation was induced. In another study, it was shown that vesicles from two different types of epithelial cells are able to induce cross-transdifferentiation of these cells [145]. Extracellular vesicles can thus be considered very promising tools for targeted long-term changes in cell properties and induction of their plasticity.

## 4. Membrane Protrusions: Cytonemes, Tunneling Nanotubes and Intercellular Bridges

In recent decades, a new type of intercellular interaction through membrane protrusions has been discovered, which provides not only direct physical contact between cells, but also spatial specificity in signal transmission. There are several types of membrane protrusions, among which cytonemes, tunneling nanotubes and intercellular bridges are distinguished.

The first works on the study of membrane protrusions investigated filopodia-actin protrusions observed on migrating cells. These studies have established that cells use filopodia to explore the environment and determine the direction of cell migration [146,147]. It was subsequently determined that the formation of filopodia is not necessarily associated with cell migration but may mediate intercellular signaling [148].

### 4.1. Cytonemes

**Figure 2:**
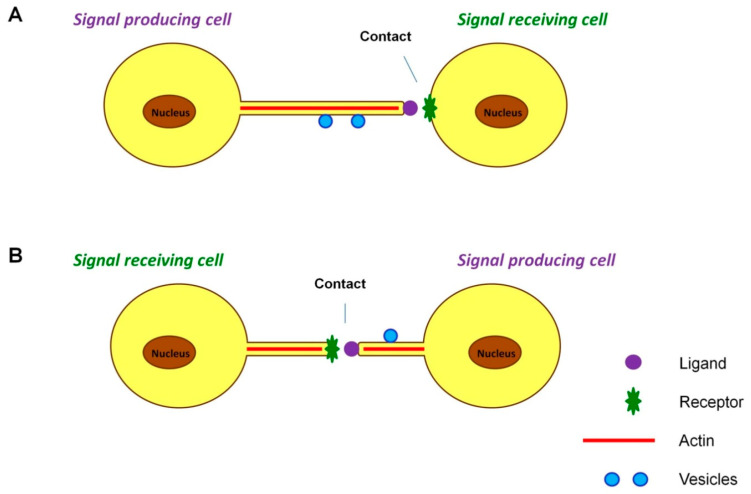
Cell signaling by cytonemes. Cytonemes are specialized closed-ended actin-based signaling filopodia. Cytoneme signaling in cells occurs due to the interaction of a ligand produced by the signaling cell localized on a growing thin membrane protrusion, which is a cytonema, with the receptor of the cell that receives the signal. (**A**) Cytonemes can grow from signal-producing cells towards receiving ones and vice versa. (**B**) The cytonemes may also be initiated by both types of cells and grow towards each other. The formation of cytonemes ends with the establishment of contact between cells. The transport of vesicles takes place on the surfaces of cytonemes, in contrast to tunneling nanotubes and intercellular bridges, where the transport of vesicles and larger cellular components can be carried out inside these membrane structures.

Filopodia of a special type include cytonemes, thin membranous protrusions of a closed type, the length of which can reach 700 microns. The formation of these structures was detected at the gastrulation stage of the sea urchin [148] and later in *Drosophila* imaginal discs [149]. In addition to actin, tubulin may also be present in the cytoskeletons of cytonemes [150]. Specific signal transduction in cells of various types that form cytonemes occurs through the interaction of signaling molecules localized on these thin membrane protrusions with the receptors of the signal-receiving cells [151,152]. In such cases, the initiation of cytonemes can occur in two directions: both from the signal-producing cell and from the signal-receiving cell (Figure 2).

The presence of cytonemes correlates with paracrine transport of major morphogens, including Notch, Wingless (Wg)/Wnt, Hedgehog (Hh)/Sonic hedgehog (Shh), Branchless (Bnl)/FGF, Spitz (Spi)/EGF and Decapentaplegic (Dpp)/BMP. Importantly, cytonemes are able to transmit signals to cells separated not only by distance, but also by other cells in the tissue [153]. Studies conducted in vitro and in vivo show that initiation of cytoneme formation can be facilitated by the expression of a morphogen. For example, overexpression of Shh, Notch Jagged ligand, FGF2 or Wnt3A in NIH 3T3 cells contributed to an increase in the incidence of cytonemes [154,155]. In a similar study in *Drosophila*, manipulation of Dpp expression affected the number and length of cytonemes in *Drosophila* imaginal discs and also interfered with the directionality of cytoneme growth [151]. The ability of morphogens to initiate the formation of cytonemes, as well as to stabilize and direct them, indicates that morphogens are likely to interact with regulators of the cytoskeleton in cytoneme-forming cells.

The Wnt/β-catenin signaling pathway is known to be involved in tissue formation and the self-renewal of many mammalian stem cells—in particular, embryonic stem cells (ESCs) [156]. This pathway is often regulated by secreted Wnt ligands in the stem cell niche [157]. Junyent et al. investigated the ability of ESC cytonemes to distinguish between those Wnt ligands that activate the Wnt/β-catenin pathway, e.g., Wnt3a, and other ones that activate β-catenin-independent pathways, e.g., Wnt5a. Using microbeads with immobilized Wnt3a and Wnt5a ligands, the authors were able to show that ESCs are able to distinguish signals from two ligands. Signal recognition was mediated by the activity of receptors that produce calcium transients, α-amino-3-hydroxy-5-methyl-4-isoxazolpropionic acid (AMPA) and kainate-glutamate in cytonemes [158]. In subsequent work, Junyent et al. demonstrated the existence of crosstalk between ionotropic glutamate receptors, such as AMPA/Kainate (iGluRs), and the Wnt pathway at the stage of initial interaction of Wnt3a with mouse ESC cytonemes. This crosstalk has been shown to persist for the entire time of ESC response to Wnt3a, with iGluRs regulating early Wnt3a recruitment, cell membrane dynamics and spindle orientation toward the source of Wnt3a during mitosis. AMPA receptors were crucial for segregation in Wnt3a-mediated asymmetric cell division. Wnt co-receptors Lrp5 and Lrp6, in concert with β-catenin, mediated dynamic interaction and spindle orientation with respect to the localized Wnt3a source. These data suggest that the Wnt-iGluR crosstalk may be involved in signaling not only in embryonic, but also in adult stem cells [159,160].

The vital role of cytonemes in the establishment of asymmetric signaling and the organization of AMPs (adult muscle progenitors) within the niche of the *Drosophila* imaginal wing disc was demonstrated in a recent study by Patel et al. [161]. This work determined that cytonemes integrate two important functions—niche-specific adhesion and fibroblast growth factor (FGF) signaling. The AMPs were able to form cytonemes containing the FGF receptor and attach to the FGF-producing wing disc niche. In turn, activation of FGF signaling in AMPs enhanced niche-specific polarity and cytoneme attachment. Such mutual interaction mediated by cytonemes can presumably play an important role in the creation and maintenance of a niche-specific asymmetric organization of stem cells. Du et al. [162], in a recent study, showed that GPI-anchored FGF stimulates the formation of cytonemes, thereby regulating its tissue-specific distribution. FGF-FGFR interactions induce bidirectional responses in cells, which in turn polarize FGF-sending and FGF-receiving cytonemes with respect to each other to enhance signaling contacts.

In addition to cytonemes, closed membrane protrusions also include microtubule-based nanotubes (MT-nanotubes) [163], while tunneling nanotubes (TNTs) and membrane nanotubes (cell tubes, bridges or ring channels) are mostly actin-based structures with an open end, although in some cases microtubules are also present in their cytoskeletons [164]. Thanks to tunneling and membrane nanotubes, the exchange and transfer of soluble cytoplasmic components, molecules, vesicles, organelles and pathogens between cells is ensured [165,166].

### 4.2. Tunneling Nanotubes

Tunneling nanotubes (TNTs) were first described in 2004 as a new type of intercellular interaction capable of organelle transfer between cells. A distinguishing feature of TNTs is the fusion of membranes of interacting cells at the site of TNT formation (in contrast to cytonemes, which establish contact between cells through the interaction of signaling molecules located on their membranes) [165]. Subsequent studies revealed a great diversity in the morphology and structure of TNTs. The length of TNTs in different cell lines varies greatly and can dynamically change depending on the motility and the distance between cells (on average, up to 120 μm for thin TNTs and up to 250 μm for thick TNTs). The diameter also varies depending on the cell type, averaging from 50 to 380 nm, while the diameter of thick TNTs reaches 700 nm and more [167,168].

One of the main proteins involved in the formation of TNTs is M-Sec. M-Sec has been shown to induce the de novo formation of numerous membrane protrusions, some of which attach to neighboring cells and subsequently form tunnel-like structures. At the same time, inhibition of M-Sec expression and blocking of M-Sec interaction with RalA and the exocyst complex led to a significant decrease in endogenous TNT production [169]. Another study suggested the role of LST1 protein in TNT formation by demonstrating that overexpression of the transmembrane form of LST1 promotes the formation of thin membrane protrusions up to 300 µm long that resemble nanotubes [170]. The LST1 protein induces the formation of TNTs by recruiting RalA GTPase to the plasma membrane and facilitates its interaction with the exocyst complex. LST1 also recruits the actin-crosslinking protein filamin and interacts with M-Sec, myoferlin and myosin. It is assumed that LST1 functions as a membrane scaffold, mediating the assembly of a multimolecular complex that controls the formation of TNTs [171].

**Figure 3:**
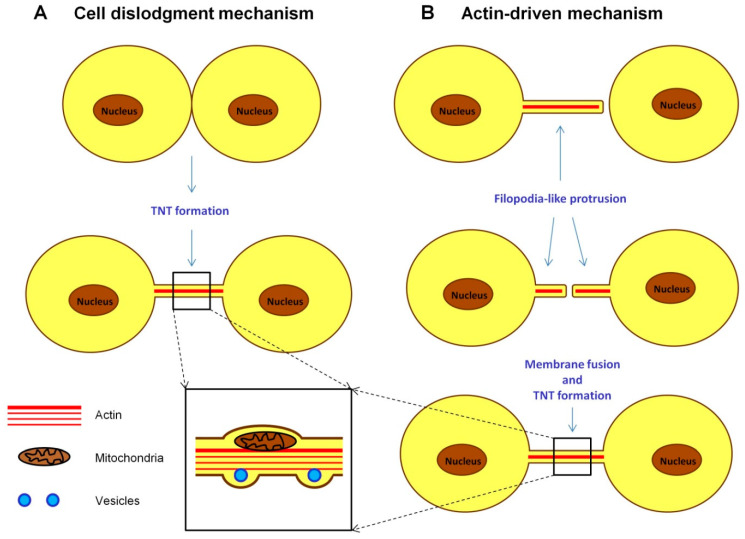
Mechanisms of tunneling nanotube formation. (**A**) Cell dislodgement mechanism involves the temporary fusion of two cells, followed by the formation of a nanotube during their separation. (**B**) Actin-driven mechanism involves the formation of filopodial protrusions by one or two cells, with their subsequent fusion. Importantly, TNT formation always ends with the fusion of the membranes of two cells, in contrast to the cytonemes, which establish contacts between cells without cell fusion. The organelles and other cellular components can be transported inside TNTs, with the diameter of TNTs increasing in the region of cargo passage.

To date, two mechanisms of TNT formation have been described (Figure 3). The first involves the formation of TNTs by temporary fusion of cells and the preservation of a thin membrane filament in the process of their separation. The second mechanism is an actin-controlled formation of a filopodia-like protrusion by one cell, followed by its fusion with another cell body or similar protrusion formed by another cell.

In both mechanisms, TNT formation is regulated by a family of small GTPases—in particular, Rac1, RalA, RhoA and Cdc42—and their downstream effectors, WASP (the Wiskott–Aldrich Syndrome protein) and WAVE (WASP-family verprolin-homologous protein), through their involvement in actin polymerization [172]. It is important to note that the signaling pathways involved in TNT formation are not necessarily identical in various cell types and may differ from those of filopodia. Thus, the activity and differential localization of Cdc42 and Rac1 are important for TNT biogenesis in macrophages [172]. However, in neurons, Cdc42, IRSp53 (Insulin Receptor Substrate 53 kDa) and VASP (vasodilatator-stimulated phosphoprotein) negatively regulate the formation of TNTs, while positively regulating the formation of filopodia. Vice versa, Eps8 (epidermal growth factor receptor pathway 8) is a positive regulator of TNT formation in neurons but inhibits the formation of filopodia in these cells [173]. A recent study using a synthetic three-dimensional matrix showed that the formation of TNTs depends on the geometry of the matrix, with the parallel arrangement of matrix fibers leading to the formation of sparser yet longer TNTs compared to those formed on cross-arranged fibers [174].

A potentially important study demonstrated recently a novel mechanism of tunneling nanotube formation by the fusion of exosomes with each other during in vitro blood–brain barrier genesis, which may be essential for bilateral signaling between adjacent brain endothelial cells [175].

**Figure 4:**
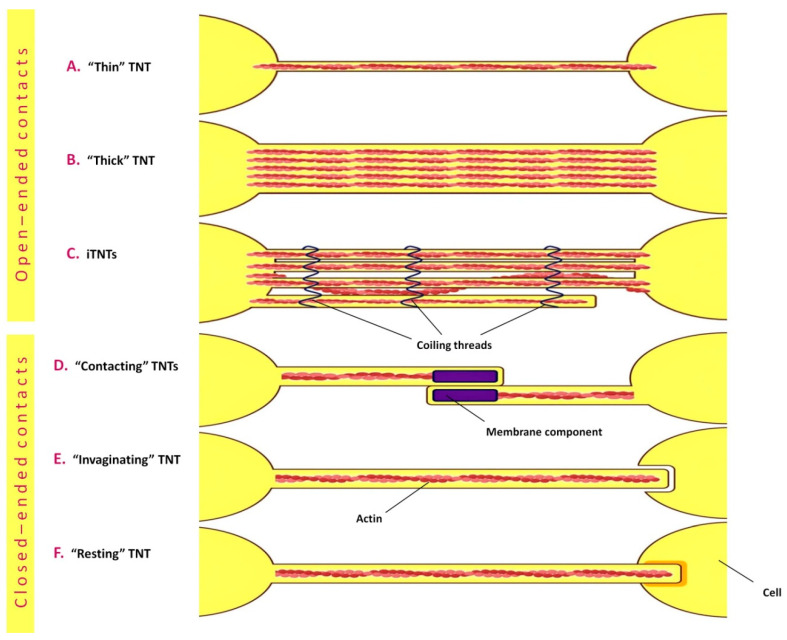
Types of tunneling nanotubes with open- and closed-ended contacts. Electron microscopy studies demonstrate that tunnelling nanotubes are quite dynamic structures that can belong both to the open-ended (**A**–**C**) and closed-ended (**D**–**F**) membrane protrusions. Scanning electron microscopy (SEM) and cryo-transmissive electron microscopy (cryo-TEM) revealed the existence of thin (**A**) and thick (**B**) TNTs. Thick TNTs are usually large in diameter but may also be represented by bundles consisting of individual tunneling nanotubes (iTNTs) that are mostly parallel to each other but sometimes may be intertwined. These individual TNTs are connected, in addition to the cell adhesion molecule N-cadherin, by coiling threads around iTNTs (**C**). Closed-ended types of TNTs presumably promote intercellular interaction through gap junctions. Serial-sectioning transmissive electron microscopy (ssTEM) revealed two closed-ended TNT types in immune cells, namely, a TNT type with two protrusions that contact in the middle, with membrane components located at the contacting tips (**D**), and single TNTs invaginating into the opposite cell (**E**). Focused ion beam scanning electron microscopy (FIB SEM) revealed the resting form of closed-ended TNTs (**F**).

In one of the recent works using real-time fluorescence microscopy, the formation of F-actin TNTs, referred to in this work as “single filopodial bridges” (SFBs), was studied [176]. Interactions of adhesion molecules on the filopodia of two cells resulted in the formation of double filopodial bridges (DFBs). Ultra-high-resolution fluorescence microscopy showed that TNTs are formed as a result of helical deformation of DFBs [176]. These results are in good agreement with data obtained somewhat earlier using correlative scanning cryo-electron microscopy (SEM) [177], which demonstrated an unusual TNT conformation that enhances their stability and plasticity, with most TNTs consisting of several individual TNTs arranged in parallel with each other or intertwined [177]. Chang et al. concluded that SFBs, DFBs and TNTs formed from them have closed ends [176]. It should be noted that one of the presumed important features of TNTs, as proposed by Gerdes et al. [178], is the continuity of the cytoplasm that makes possible the transport of organelles along TNTs. Nevertheless, in a number of studies, TNTs are described as membrane protrusions with closed ends [179,180,181]. Thus, the question of TNT structure and organization remains open today, probably reflecting their dynamic nature. As a consequence of the above TNT features, at least six TNT types with open- and closed-ended contacts can be distinguished (Figure 4).

### 4.3. Intercellular Bridges

**Figure 5:**
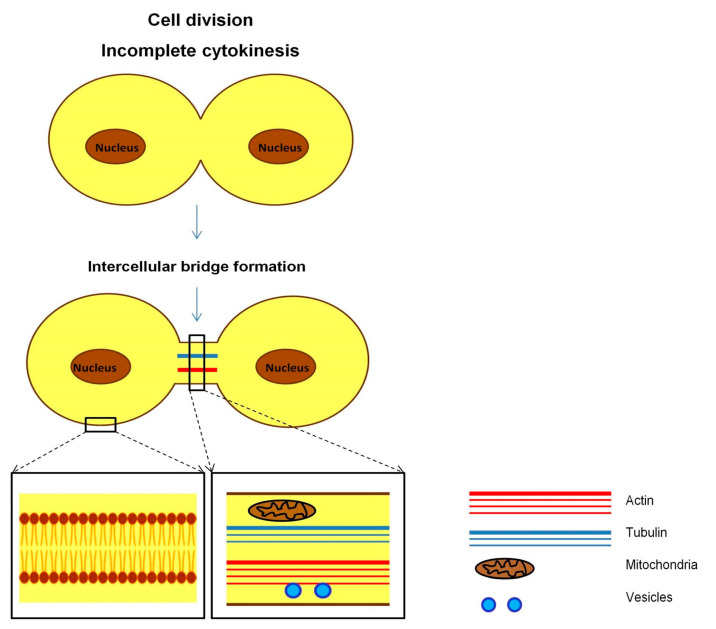
Mechanism of intercellular bridge formation. Intercellular bridges form upon completion of karyokinesis, as a result of incomplete cytokinesis. These structures represent open-ended membrane protrusions, which are shorter but have a much larger diameter compared to TNTs. The transport of organelles and other cellular components inside intercellular bridges occurs unhindered and freely, without increasing the diameters of intercellular bridges in the region of cargo passage.

Intercellular bridges, or ring channels, are structures similar in appearance to cytonemes and TNTs and may be up to 350 µm long [182]. Intercellular bridges were first noticed in the study of the differentiation of cat spermatids [183], and today these structures continue to be observed mainly in germ cells [184,185]. Like TNTs, intercellular bridges are involved in molecular and organelle transport and Ca^2+^ signaling [186,187]. However, the mechanism of formation of intercellular bridges is different from other actin-based membrane protrusions and is a result of incomplete cytokinesis (Figure 5). If the cytokinesis of somatic cells ends with the formation of independent daughter cells, the completion of cytokinesis in germ cells is characterized by the formation of stable intercellular bridges that connect daughter cells. Intercellular bridges in mammals thus facilitate the formation of syncytium from hundreds of clonally derived germ cells, while the interruption of syncytium formation leads to defects in spermatogenesis [188,189,190].

The intercellular bridges, compared to other filopodial protrusions, are larger in diameter, varying from 0.2 to 10 µm, but are usually shorter. They include actin and the scaffold protein anillin, while tubulin was found only in the male germ line [184]. In *Drosophila melanogaster*, intercellular bridges formed between female germ cells are called annular canals, which facilitate the transfer of nutrients and cytoplasmic components into the developing oocyte from other cells that subsequently die [191,192]. Despite the fact that the functions of intercellular bridges and TNTs are broadly similar, these formations clearly represent different structures.

### 4.4. Membrane Protrusions as Mediators of Pathogenesis and Candidates for Targeted Therapy

Membrane protrusions, similar to extracellular vesicles, are involved in the various types of pathogenesis. Bacterial and viral invasions initiate the formation of cytonemes and TNTs, which pathogens use to penetrate cells and begin reproduction [193,194]. In addition to bacteria and viruses, fungal spores, prions, organelles and other molecules can be transported via TNTs [195,196,197].

Small GTPases were shown to be involved in many cases of infection. Thus, Gram-negative Shigella bacteria induce Cdc42 activation [198], while the Salmonella IpgB1 protein functions as a RhoA GTP-binding protein, also causing actin polymerization in host cells [199]. Intracellular invasion of *Mycoplasma hyorhinis*, triggering Rac1 activation, induces the formation of TNTs in mammalian cells, which contribute to the further spread of these bacteria [194]. The cyanobacterial macrolide tolitoxin, which inhibits actin polymerization and thus significantly reduces numbers of TNTs, can therefore serve as a valuable tool for the treatment of disease spread through TNTs [200]. In addition to tolitoxin, the formation of TNTs is blocked by cytochalasins [201], as well as cytarabine (cytosine arabinoside), which in this capacity acts as an inhibitor of intercellular transmission of human T-lymphotropic virus type 1 (HTLV-1) [202].

Studies on the role of TNTs in oncogenesis deserve special attention. Tunneling nanotubes appear to be important factors in the interactions between tumor cells and macrophages, which may lead to tumor progression and the formation of metastases [203]. Recently, Lee et al. [204] showed that media conditioned by M0 and M1, but not M2, macrophages strongly stimulate the formation of TNTs in human pancreatic cancer cells (PANC-1), which correlates with increased PANC-1 mobility and epithelial-mesenchymal transition; transport of mitochondria and lysosomes via TNTs also occurs. TNTs may play a primary role in the earliest phases of both physiological and tumor angiogenesis in the brain [205]. Turner et al. showed that overexpression of the BRAF V600E mutation promotes changes in the cell phenotype and induces the formation of tunneling nanotubes that accumulate lipid droplets [206]. In a work on TNT-mediated resistance of MCF-7 breast cancer cells to chemotherapy, Kato et al. [207] showed that cytotoxic doses of the chemotherapeutic agent 5-fluorouracil (5-FU) induced the formation of TNTs through which the exchange of cellular components—in particular, mitochondria—occurred. The treatment of MCF-7 cells with cytochalasin-B inhibited the formation of TNTs and enhanced 5-FU cytotoxicity. The results of Saha et al. [208] demonstrated the transfer of mitochondria from immune cells to tumor cells in an aggressive breast cancer model. Inhibition of this TNT-mediated mitochondrial hijacking resulted in improvement in antitumor outcomes.

The work by Bouhaddou et al. [209] established that infection of Vero E6 cells with SARS-CoV-2 substantially changed the phosphorylation of host and viral proteins and induced a dramatic increase in filopodial protrusions, which were significantly longer and more branched compared to uninfected cells. In addition, these filopodia contained budding virus particles, which may be important for the spread of SARS-CoV-2 in epithelia [209]. Merolli et al. [210] showed the presence of active intercellular interactions in Vero E6 cells infected with mNeonGreen-expressing SARS-CoV-2. With the aid of scanning helium microscopy, long TNTs that connected infected cells over submillimeter distances were observed; the formation of syncytia and abundant production of extracellular vesicles were also documented.

TNTs also play an important role in the pathogenesis of HIV (HIV). A number of studies have shown a correlation between HIV infection and the presence of TNTs in HIV-infected cells [211,212,213,214,215]. Hashimoto et al. showed that HIV-1 promotes the formation of TNTs through viral Nef and cellular M-Sec proteins [212]. Subsequently, M-Sec was shown to mediate rapid and efficient intercellular transmission of HIV-1 at an early stage of infection by increasing the formation of TNTs [214]. Bertacchi et al. [215] recently confirmed the importance of TNTs in HIV infection of dendritic cells (DCs) and further demonstrated that inhibition of TNT formation by cytarabine (AraC) reduced infection in a DC culture and a DC/CD4^+^ T-cell coculture.

Based on the above results, it can be concluded that TNTs and cytonemes are very promising therapeutic targets for the development of drugs that block the transmission of viral, bacterial and oncological diseases through these mechanisms.

### 4.5. Membrane Protrusions as Emergency Care for Cells

Although membrane protrusions may play a negative role in pathological processes, their main role in the body is, of course, the opposite. Potential methods for membrane-protrusion-mediated delivery of subcellular components for the repair of damaged cells are currently being actively investigated. The greatest attention in this regard is being paid to MSCs. Zhao et al. [216] showed, for example, that MSCs promote cell survival in acute kidney injury. The kidney recovery occurred due to the transfer of subcellular components from MSCs to the kidney cells through both the formation of TNTs and the secretion of extracellular vesicles.

Healthy mitochondria are essential for ensuring normal energy metabolism and providing cells with energy through oxidative phosphorylation [217]. Currently, more and more evidence is accumulating for the importance of mitochondrial transfer for the restoration of the functional activity of damaged or depleted cells. In particular, the mechanism of the antimicrobial action of MSCs in acute respiratory distress syndrome was recently revealed, which is based on increased phagocytic activity through the transfer of mitochondria from MSCs to macrophages using TNTs [218]. The work by Jiang et al. showed that in a co-cultivation system, mitochondria are transferred from MSCs to corneal cells via TNTs [219]. The formation of the latter is significantly enhanced under oxidative stress and is mediated by the NF-κB/TNFαip2 signaling pathway. MSC transplantation also had a significant positive effect in a rabbit corneal alkali burn model [219].

The work by Feng et al. [220] studied the ex vivo formation of TNTs between bone marrow MSCs and human umbilical vein endothelial cells (HUVECs) and showed the existence of mitochondrial transfer from MSCs to damaged endothelial cells, which preserved their aerobic respiration and protected from apoptosis. Similar mechanisms of mitochondrial protection may be involved, in particular, in recovery after ischemic stroke [221]. Through a similar mechanism, MSCs from Wharton’s jelly can exert a therapeutic bioenergetic effect on cells with mitochondrial disorders, such as the syndrome of mitochondrial encephalomyopathy with lactic acidosis and stroke-like episodes (MELAS) [222]. Studies performed on astrocytes subjected to ischemic injury showed that overexpression of the Miro1 gene in MSCs increases the efficiency of transport of mitochondria from them to damaged astrocytes, resulting in the restoration of the bioenergetics of recipient cells and stimulation of their proliferation [223]. More recently, Yang et al. [224] reported that Miro1-mediated transfer of mitochondria from MSCs via TNTs restores the function of nucleus pulposus cells (NPCs) after mitochondrial dysfunction induced by rotenone treatment. This finding lays a foundation for the future development of efficient therapies for intervertebral disc degeneration.

It is known that cancer patients undergoing chemotherapy often develop cognitive impairment, including memory loss and decreased attention [225]. Boukelmoune et al. [226] observed the transfer of mitochondria from MSCs to neural stem cells (NSCs) exposed to neurotoxic effects of the chemotherapeutic agent cisplatin. Mitochondrial transfer increased the survival of damaged NSCs, whereas blocking the formation of actin-based intercellular structures led to the inhibition of this transfer and eliminated the positive effect of MSCs on NSC survival [226].

Importantly, the transfer of mitochondria can also occur with the participation of extracellular vesicles. Thus, Korpershoek et al. [227], in addition to the transfer of mitochondria from MSCs to chondrocytes using direct intercellular contacts and TNTs, also established a third mechanism of mitochondrial transfer via extracellular vesicles, demonstrating the presence of mitochondria in EVs. In a similar study, Thomas et al. [228] reported that MSCs are able to package functional mitochondria into extracellular vesicles that can be transferred to chondrocytes without direct intercellular interactions. A new mechanism of acquired chemoresistance of triple-negative breast cancer cells by the EV-mediated transfer of mitochondria was shown by Abad et al. [229]. On the other hand, an interesting study by Crewe et al. [230] demonstrated that the injection of EVs released by stressed adipocytes and containing oxidatively damaged mitochondrial particles may have, contrary to expectations, a positive effect and alleviate cardiac ischemia in mice, representing a first example of interorgan mitohermesis in vertebrates. According to Thomas et al. [228], mitochondria, which are part of extracellular vesicles, are functionally active, while Zorova et al. [231] stated that mitochondria in extracellular vesicles are inactive. However, even in the latter case, it is quite probable that, after entering the target cells, mitochondria can restore their functional activity.

Finally, one should mention a highly unexpected mechanism of intercellular transfer of subcellular components discovered quite recently. Lanna et al. [232] showed that antigen-presenting cells are able to shed their telomeres, pack them into extracellular vesicles and transmit them through immunological synapses to certain classes of T cells. This protects the latter from premature aging associated with subsequent clonal expansion and promotes immunological memory. It is possible that a similar mechanism of telomere donation may also exist in some other cell systems.

Based on the above results, it can be concluded that the transfer of mitochondria, as well as other subcellular components, through membrane protrusions and extracellular vesicles is a quite promising new avenue for the development of regenerative therapeutic strategies.

## 5. Concluding Remarks

The studies reviewed above shed light on the various manifestations of non-classical intercellular communications taking place in living organisms. It is becoming increasingly clear that these communications and interactions play ubiquitous and paramount roles in a variety of biological processes. However, the magnitude and full range of their involvement is yet to be established. How these non-classical interactions compare with the classical ones in terms of their biological effects is also a crucial question awaiting clarification.

An important point regarding non-classical communications, whether via extracellular vesicles or membrane protrusions, is that they are driven by structural and functional molecular ensembles that involve simultaneously many actors belonging to different classes of macromolecules. This makes these interactions much less defined and amenable to molecular analysis, while rendering mechanistic studies of their action more complicated and ambiguous. On the other hand, numerous experiments in biology have demonstrated clearly that the combined action of several selected factors is usually more effective compared to the action of a single factor. Similarly, the vast medical practice demonstrates that rational combinations of several drugs or therapies are usually accompanied by better and more durable outcomes compared to monotherapies. Given these considerations, one may predict that the development of approaches using non-classical intercellular communications and capitalizing on the distinctive advantages of their action modes will be one of the most important avenues of further research and development in biomedicine. In particular, it seems quite plausible that these interactions may be very successfully used in novel therapeutic regenerative strategies that will be able to overcome problems that accompany the use of stem cells in medicine.

## Data Availability

Not applicable.
